# Improvement of the country safe community program in view of changing the pattern of traffic accidents in the under-19 age group in Iran 

**Published:** 2019-08

**Authors:** Gholamreza Masoumi, Arezoo Dehghani

**Affiliations:** ^*a*^Emergency Medicine and Health in Emergencies and Disasters, Trauma and Injury Research Center, Iran University of Medical Sciences, Tehran, Iran.; ^*b*^School of Public Health and Safety, Shahid Beheshti University of Medical Sciences, Tehran, Iran.; ^*c*^Health in Emergency and Disaster Research Center, University of Social Welfare and Rehabilitation Sciences, Tehran, Iran.

## Abstract

**Background:**

Safe community is based on four principles: safety belief, knowledge of the proper use of safety devices, access to safety requirements and respect for the rules, and safe driving is one of its axes. According to the World Health Organization s annual report for 2015, more than 1.25 million People die because of traffic accidents worldwide, and the eighth cause of death in the world is the leading cause of death in the age group of 15-15 years. This study was carried out to promote the safe community based on traffic accident analysis in the age group under 19 years old in Iran.

**Methods:**

This study was conducted through document review, reassessment of documents, data from the World Health Organization, Death and Immortality of Iran, and the Forensic Medicine Organization.

**Results:**

According to the statistics, in 2016, there was a change in the pattern of injuries in adolescence, and the first three priorities were changed to traffic accidents (32%), fall (16%), poisoning and violence (5%). Is. The study of the 10-year trend of traffic accidents in three types of pedestrians, motorcyclists and motorcycles shows that the age group of 15-19 years old males in all three levels is in the number of injuries. In the traffic accident cases, the pattern of injuries has changed. Despite the fact that 34.5% of the victims of the incident belonged to the age group of 19 to 15 years old, it was ranked second in the age group of 19-15 years old. The major causes of driving accidents in the age group of 19-15 years were not having a driving license, mental and emotional state, non-use of seat-belts, imagine the ability to control a car or engine, dramatic movements during driving and Lack of attention to driving directions which could be prevented.

**Conclusions:**

All organizations involved in traffic safety and the safe community should focus on providing cultural, educational, and empowering age groups with the explanation of the areas of technical, scientific, educational and research services and the design of safety promotion interventions. Preventive strategies should be employed at three primary, secondary and tertiary levels.

**Keywords:**

Safe community, Traffic Accidents, Prevention, Child and Adolescent

